# Levels of Chemical Toxicants in Waterpipe Tobacco and Waterpipe Charcoal Solid Waste

**DOI:** 10.4236/jep.2021.1211054

**Published:** 2021-11-26

**Authors:** Jason R. Hsieh, Megan L. Mekoli, Ronald L. Edwards

**Affiliations:** Office of Science, Center for Tobacco Products, U.S. Food and Drug Administration, Calverton, MD, USA

**Keywords:** Harmful and Potentially Harmful Constituents, Charcoal, Waterpipe, Metals, Tobacco Product Waste

## Abstract

This work provides insights on waterpipe tobacco and waterpipe charcoal as potential sources of environmental toxicants. Selected harmful and potentially harmful constituents (HPHCs) from ten U.S. commercial waterpipe tobacco filler products (before and after electric heating) and five waterpipe charcoal products (before and after burning) were investigated. The differences in quantities of HPHCs between the evaluated products appear to be affected by raw material properties and/or the manufacturing processes involved in product production. Trace metal quantities in waterpipe tobacco and charcoal products were observed after heating or burning conditions compared to unheated or unburned conditions, which could impact the environment through the generation of toxic tobacco product waste. This study demonstrates that waterpipe tobacco and waterpipe charcoal contain substantial quantities of benzo[a]pyrene (B[a]P) and trace metals (*i.e*., selenium, arsenic, cadmium, chromium, cobalt, lead, nickel) before use and that extensive and varied changes in trace metal quantities take place as a result of heating, and more studies are needed to estimate the magnitude of the environmental impact of waterpipe tobacco use.

## 1. Introduction

The use of waterpipe (WP) tobacco (also known as hookah, maassel, narghile, argileh, shisha) and WP charcoal as a heating source has increasingly spread among youth and young adults worldwide [1] [2] [3] [4] [5]. WP tobacco smoking is often incorrectly perceived as less harmful, less addictive, and a safer alternative to combustible cigarette smoking [6]. The World Health Organization (WHO) estimated that a single session of WP smoking could release as much as 100 – 200 times the smoke volume of a single cigarette [7] [8]. Several studies have investigated the harmful and potentially harmful constituents (HPHCs) in WP tobacco smoke, such as polycyclic aromatic hydrocarbons (PAHs), carbon monoxide (CO), nicotine, and other toxicants (tar) produced from WP smoking [9] [10]. However, few studies have assessed the quantities of HPHCs remaining in WP tobacco and WP charcoal product wastes after use, or in WP tobacco separate from WP charcoal. The U.S. Food and Drug Administration (FDA) received authority to regulate the manufacturing, distribution, and marketing of tobacco products in the United States through the Family Smoking Prevention and Tobacco Control Act in 2009 [11]. FDA further extended its tobacco regulatory authorities to other tobacco products (Deeming Rule), including WP tobacco and WP charcoal, in 2016 [12]. This study will inform FDA’s efforts to fulfill the National Environmental Policy Act (NEPA) which requires that U.S. federal agencies evaluate the environmental impacts of proposed actions such as reviewing applications for marketing authorization and developing product standards to improve public health.

**Figure 1.**
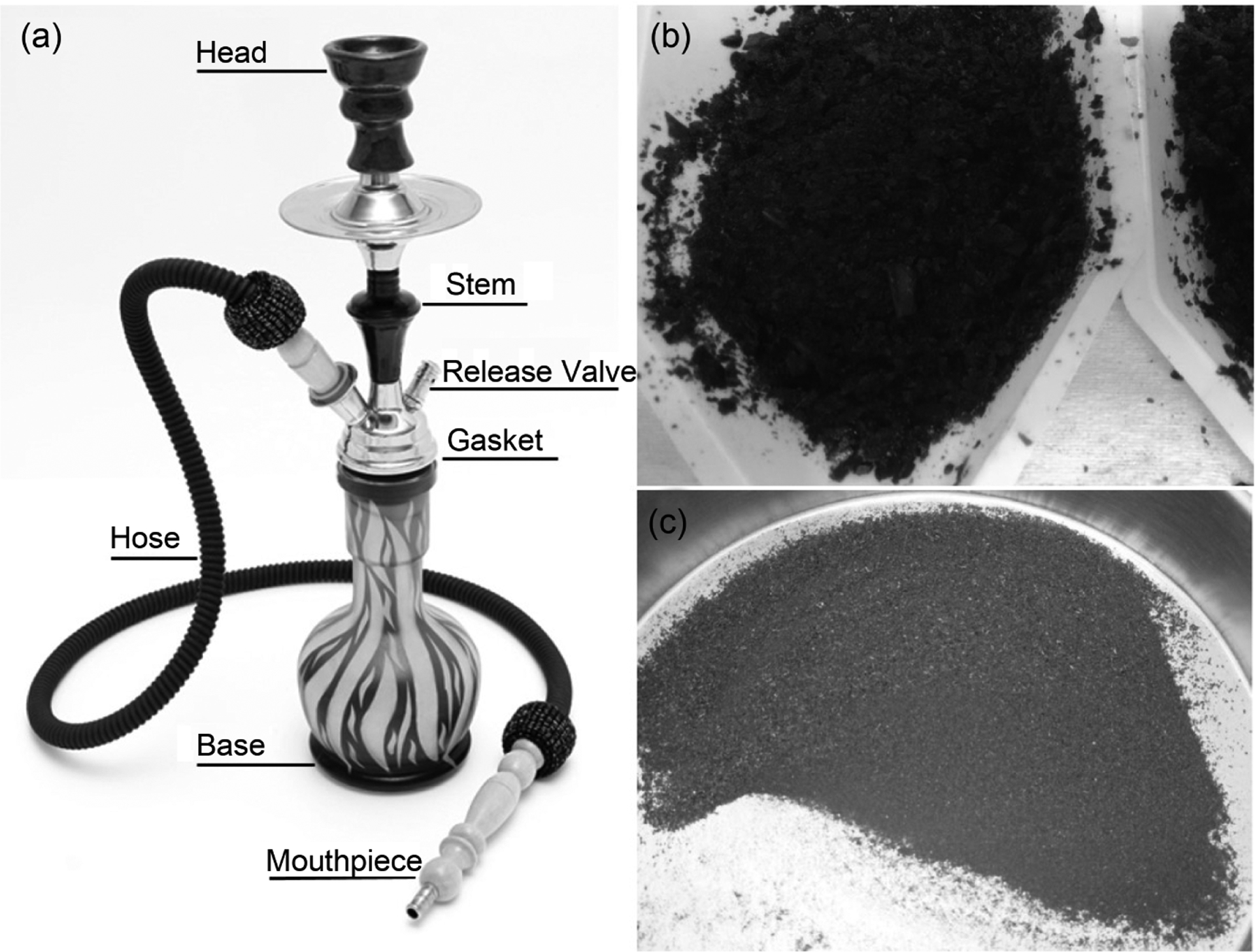
(a) Representative schematic diagram of a WP apparatus; (b) Representative product images of homogenized WP tobacco before heating; (c) Homogenized WP charcoal before burning.

A WP setup consists of a head, stem, gasket, base, release valve, hose, and mouthpiece (Figure 1(a)). For typical WP smoking, a mixture containing WP tobacco and other additives is placed in the head, covered with perforated aluminum foil, and indirectly heated using smoldering charcoal. The produced smoke passes through the base, which is partially filled with liquid, and then inhaled by the user through a hose (with mouthpiece) connected to the WP base [13]. Although water is often used to fill the base, it has been reported that additional flavors can be introduced by substituting the water with milk, wine, fruit juice, or energy drinks [14] [15]. Users often believe [16] that liquid “filtration” in the WP apparatus can efficiently remove excess HPHCs, such as toxic metals, produced from WP tobacco and WP charcoal. However, Al-Kazwini *et al*. determined that only about 3% of the total metal is removed from smoke through the water base of the WP apparatus [17].

Flavored WP tobacco is often used in WP smoking; flavored WP tobacco is a complex mixture [18] composed of approximately 30% tobacco and 70% non-tobacco ingredients and may contain glycerin, molasses, honey, sugar, fruit pulp, other flavorings (e.g., mint, coconut, peach, cola, bubble gum, grape), and preservatives [19]. Aromatic smoke produced from flavored WP tobacco may be particularly appealing to youth [20]. Several studies have investigated human health diseases (e.g., lung disease, oral cancer) associated with WP smoking [21] [22] and exposure to high concentrations of heavy metals is known to cause adverse effects on humans [23]. Specifically, the tobacco plant is capable of trapping and accumulating heavy metals [24], including cadmium (Cd), zinc (Zn), copper (Cu), iron (Fe), and lead (Pb), which are also commonly found in other tobacco products [25].

WP charcoal is used as a heating source in WP smoking and can be a potential source of contamination to the user and the environment. WP charcoal temperature can reach close to 450°C compared to 900°C in a burning cigarette [26]. Several studies have investigated the formation of HPHCs as a function of temperature in other tobacco products [27] [28] [29] [30]. WP charcoal is commonly produced from one of three sources: coconut husks, woods, or sugar cane stalks [31]. Heavy metals(e.g., zinc [Zn], Pb, Cd, Cu, nickel [Ni]) can be absorbed by the roots of plants [32], and the quantities of heavy metals in charcoal are dependent on the origin of the wood, type of wood, soil type and quality, and manufacturing processes [31]. Upon completion of a smoking session, WP tobacco and charcoal residues are often disposed into the environment, which adds to overall environmental contamination. Although the impact of environmental contamination of tobacco products such as cigarette butts has been investigated extensively [33] [34] [35] [36] [37], research on WP-associated wastes and their environmental impact is more limited. This work investigates WP tobacco residue and WP charcoal waste containing HPHCs such as metals (*i.e*., arsenic [As], Cd, cobalt [Co], chromium [Cr], Ni, Pb, selenium [Se]), PAHs (e.g., B[a]P), carbonyls (e.g., acrolein, formaldehyde [FA]), volatile organic compounds (VOCs) (e.g., benzene, isoprene), phenols (e.g., catechol, phenol), and tobacco-specific nitrosamines (TSNAs) (e.g., N-nitrosonornicotine [NNN], 4-(methylnitrosamino)-1-(3-pyridyl)-1-butanone [NNK]); these HPHCs are selected based on the commonality in WP tobacco and WP charcoal smoke, and they are on the FDA established list of 93 HPHCs in tobacco products and tobacco smoke.

## 2. Materials and Methods

### 2.1. Preparation of Heated Tobacco and Burned Charcoal Samples

A smoking machine (Hawktech FP2000 or Borgwaldt Shisha Smoker) operating under the Beirut puffing regimen [38] (puff volume: 0.530 L, puff duration: 2.6 sec, interval between puffs: 17 sec, 57 min smoking session for total 171 puffs) was used to smoke a commercially available WP with one of ten selected brands of tobacco with different flavors. All WP tobacco and WP charcoal samples were purchased from online vendors and selected for their random flavors and additives and stored at room temperature. Ten WP tobacco brands with different flavors (chocolate and mint for T1, blueberry and mint for T2, orange and cherry for T3, passion fruit for T4, blueberry and mint for T5, blueberry and mint for T6, apple for T7, peach for T8, watermelon for T9, and orange for T10) and five WP charcoal brands with different materials and shapes (beech wood and disc shape for C1, coconut husks and cube shape for C2, instant and disc shape for C3, quicklight and disc shape for C4, natural and finger shape for C5) were investigated (N = 7 per brand). All WP tobacco brands were traditional tobacco except the T9 brand, which was a nicotine gel.

All WP tobacco and WP charcoal samples were weighed at the beginning of each machine smoking session. The smoking machine pulled air through a quantity of tobacco (10 g) in a head. The WP tobacco was then indirectly heated by an in-house electric heater, achieving similar temperatures as commercially available heaters (360°C) [39] [40]. The resulting smoke was pulled down the stem (Figure 1(a)), bubbled through the water reservoir containing 18 MΩ deionized water in the base (enough water to cover 30 mm of the stem, approx. 1.5 L), and passed through filters placed in-line at the junction between the base and the tubing to collect total particulate matter (TPM). For charcoal sample preparation, charcoal was placed on an electric burner (for lighting charcoal) for 100 sec before placing it onto the ceramic WP head wrapped with perforated foil (18 holes). The smoking session was initiated and allowed to run undisturbed. At puff 99, the remaining charcoal (half of a charcoal) was lit and allowed to burn for an additional 100 sec before being placed onto the aluminum foil at puff 105. The smoking session was complete at puff 171 (57 min). Heated WP tobacco and burned WP charcoal were allowed to cool to room temperature, then transferred to a muffled glass container, and stirred until the mixture was homogenous. Homogenized samples were stored in sealed glass jars before testing. The consumed WP tobacco and WP charcoal masses were determined gravimetrically.

### 2.2. Homogenization of Unheated Tobacco and Unburned Charcoal Samples

All tobacco samples were aerated, cleaned of debris (removal of stems, twigs greater than 1 cm in length, whole leaves greater than 4 cm^2^, and stems) and then homogenized (approx. 100 g) using gloved hands (nitrile). After homogenizing, each tobacco sample was added to a 40 oz stainless steel carafe, chilled with dry ice, and blended (Waring model 33BL79, 120 volts/3amps) for three minutes or until particles with size approximately 4 mm were obtained visually (Figure 1(b)). After blending, any dry ice and excess moisture were allowed to evaporate to completion in a fume hood. All charcoal samples were placed into a plastic bag (9” × 12” heavy-walled, zip-seal) and crushed with a rubber mallet inside of a fume hood. All crushed charcoal samples were sieved (No. 30 sieves, 600 μm mesh) to yield a uniform particle size (Figure 1(c)). Charcoal samples intended for elemental analysis were treated differently from tobacco samples (no homogenization or sieving) to avoid possible contamination of the elements of interest from the stainless-steel carafe or metal sieve.

### 2.3. GC-MS Analysis of VOC Compounds

Purge-and-trap gas chromatography/mass spectrometry (GC-MS, [Agilent 6890 and 5973]) was used to identify and quantify isoprene and benzene in WP tobacco samples using established methods [41] [42] [43] [44]. VOCs in solid samples were introduced into the GC (Agilent 6890 GC with an Agilent 5973 Mass Selective Detector) by purge-and-trap techniques. An inert gas was bubbled through an aqueous phase to purge the VOC analytes from the aqueous phase into the gas phase. For low-level samples (samples with least preparation), laboratory reagent-grade water was used for the aqueous phase. Medium-level samples (samples requiring additional extractions or dilutions) were initially extracted in methanol (10 mL), and an aliquot (~3 – 4 mL) was diluted in 20 mL of laboratory reagent water, creating the sample aqueous phase. The gas was swept through the sorbent trap (VOCARB 3000, Carbopack B/Carboxen 1000 & 1001, 10 cm Carbopack B, 6 cm Carboxen 1000, 1 cm Carboxen 1001), where the volatile components were retained. After purging was complete, the sorbent trap was heated and backflushed with inert gas to desorb the components onto a non-polar fused silica capillary chromatographic column (Restek RTX-DHA 50 chromatographic column, 50 m × 0.20 mm internal diameter fused silica capillary column with a 0.5 μm bonded phase). The desorbed components were then separated via capillary GC and identified and quantified using electron ionization (positive and negative) mass spectrometry (MS) in the full-scan mode. Deuterated benzene, deuterated toluene, and deuterated ethylbenzene were used as internal standards, and 1,4-difluorobenzene, deuterated chlorobenzene, and deuterated 1,4-dichlorobenzene were used as surrogate samples.

### 2.4. HPLC Analysis of Carbonyl Compounds

FA and acrolein in WP tobacco samples (1 g of solid sample each) were extracted with 20 mL of extraction solution (64.3 mL of 1.0 N NaOH, 5.7 mL of glacial acetic acid, 930 mL of deionized water, at pH 4.9). The sample extract was decanted through a glass fiber filter paper in a 200 mL glass jar, then 10 mL of filtered extract was diluted with 90 mL of deionized water, 4 mL of 1 M concentrated citrate buffer (80 mL of 1 M citric acid and 20 mL of 1M sodium citrate, at pH 3), and 6 mL of 2,4-dinitrophenylhydrazine (DNPH) solution (70% w/w in acetonitrile) in a 200 mL glass jar with a screw cap. The combined solution was shaken (90 rpm) on an oscillating incubator at 40°C for 1 hour. A 10 mL of saturated NaCl solution was added into the combined solution and shaken well, and then the combined solution was transferred onto a C18 solid-phase extraction (SPE) cartridge (Thermo Scientific). A vacuum pump was used to ensure that all the solution was drawn through the cartridge at a rate of 3 – 5 mL/min. The solution was passed through the C18 SPE cartridge with 9 mL of acetonitrile. The eluent was collected and analyzed using high-performance liquid chromatography (HPLC, Shimadzu Nexera system with a CBM-20A controller, DGU-20A3R degassing unit, DGU-20A5R degassing unit, LC-30AD pump, SIL-30AC autosampler, CTO-30A column oven) with an ultraviolet detector (SPD-20AV) using established methods [45] [46] [47].

### 2.5. GC-MS Analysis of B[a]P Compound

B[a]P in unheated and heated WP tobacco, and unburned and burned WP charcoal samples (1 g of solid sample each), was extracted by sonication (at 30 or 50°C for 2 hours) using 15 mL of toluene. Each extract was spiked with deuterated perylene as an internal standard and passed through an SPE cartridge using 10 mL of hexane. Each eluent was concentrated to dryness using a nitrogen evaporator (Organomation N-evap Analytical Evaporator, model 111) at room temperature, and brought back up to 100 μL with acetonitrile (ACN), prior to analysis using GC-MS (Agilent 6890N GC with an Agilent 5975MS), operated in selected ion monitoring mode (SIM), using established methods [10] [48] [49] [50]. The quantitation ion (m/z) and confirmation ion (m/z) were 252 and 253, respectively.

### 2.6. LC-MS/MS Analysis of TSNA Compounds

NNN and NNK in unheated and heated WP tobacco samples (1 g of solid sample each) were extracted using 40 mL of 100 mM ammonium acetate. Deuterated form of NNN-d_4_ and NNK-d_4_ were added (160 μL of a 5 ng/μL solution into 40 mL extraction solvent) as internal standards, and the extract was mixed well using a rotary shaker for 1 hour at 250 rpm. One mL of each extract was filtered with a 0.45 μm GHP (hydrophilic polypropylene membrane) syringe filter on a 5-mL BD (Becton Dickinson) syringe and added into autosampler vials, followed by analysis using LC-MS/MS (Waters ACQUITY HPLC and Waters Xevo TQ MS) in positive electrospray ion mode, multiple reaction monitoring (MRM) mode, and with an analytical column (Phenomenex Gemini C18, 5μ 110A, 50 × 2 mm) [49] [51] [52]. A gradient consisting of 0.1% acetic acid in water (solvent A), and 0.1% acetic acid in methanol (solvent B) at a constant flow rate of 100 μL/min was used. The solvent gradient program was as follows: 0 min, 40% B; 0.5 min, 40% B; 5.0 min, 65% B; 5.5 min, 100% B; 6.0 min, 100% B; 6.1 min, 30% B; 8.0 min, 30% B. The injection volume was 20 μL. The column temperature was maintained at 40°C. MS/MS transitions (m/z) for NNN were as follows: 178 → 148 (quantitation); 178 → 120 (confirmation); 182 → 152 (NNN-d_4_ internal standard). MS/MS transitions (m/z) for NNK were as follows: 208 → 122 (quantitation); 208 → 79 (confirmation); 212 → 126 (NNK-d_4_ internal standard).

### 2.7. LC-FLD Analysis of Phenolic Compounds

Phenol and catechol in unheated and heated WP tobacco samples (1 g of solid sample each) were extracted using 50 mL of 1% (v/v) acetic acid in water and mixed using a shaker (New Brunswick Scientific Co., model G-3.3) for an hour. The liquid layer of extraction solution was decanted into a 50 mL polypropylene centrifuge tube and centrifuged for 2 min at 2000 rpm. After centrifugation, 1 mL of sample extract was filtered through a 0.45 μm nylon syringe filter into an LC vial for analysis using liquid chromatography with a fluorescence detector (LC-FLD) [53]. Analytes of interest were separated using a column (Phenomenex; 150 mm × 3.0 mm, 3 μm particle size Devosil 3u RP-Aqueous C30 140A). A gradient consisting of 1.0% acetic acid in water (solvent A) and 0.1% acetic acid in acetonitrile (solvent B) at a constant flow rate of 0.55 mL/min was used. The solvent gradient program was as follows: 0 min, 3% B; 3 min, 3% B; 5 min, 19% B; 15 min, 19% B; 17 min, 85% B; 19 min, 85% B; 21 min, 3% B; 24 min, 3% B. The oven temperature was maintained at 40°C, the injection volume used was 10 μL, and the excitation and emission wavelengths used were 274 nm and 298 nm, respectively.

### 2.8. ICP-MS Analysis of Trace Metals

Seven selected trace metals (As, Cd, Co, Cr, Ni, Pb, and Se) were measured in unheated and heated WP tobacco samples (0.1 g of solid sample each) and in unburned and burned WP charcoal (0.5 g of solid sample each). Each sample was placed in a clean, dry microwave digestion vessel with 10 mL of concentrated nitric acid. Microwave digestion was accomplished by ramping the temperature and digesting the samples (CEM Corporation Xpress); the samples were ramped to a temperature of 170°C ± 5°C over 10 min and held at a constant temperature for 10 – 20 min) [54]. After microwave digestion, samples were allowed to cool. The cooled sample extract was placed into a 50 mL conical vial and brought to a final volume of 50 mL using 2% aqueous nitric acid. A solution containing 100 μg/L of scandium (Sc), 50 μg/L of indium (In), 50 μg/L of terbium (Tb), and 50 μg/L of yttrium (Y) in 1% nitric acid was used as an internal standard. All extract samples are analyzed using inductively coupled-plasma mass spectrometry (ICP-MS, Perkin Elmer Elan with a Dynamic Reaction Cell [DRC-e]) [54] [55].

## 3. Results

### 3.1. HPHCsin WP Tobacco

Table 1 and Table 2 show nine selected HPHCs measured in heated and unheated WP tobacco (*i.e*., acrolein, benzene, isoprene, catechol, phenol, FA, B[a]P, NNK, and NNN). All WP tobacco analyses included a blank (no solid matrix) and a control sample (1 g of sand as a solid matrix). All HPHCs quantities were corrected to account for solvent blanks. Benzene was not detected above the limit of quantification (LOQ) (56 ng/g) in all ten brands of unheated WP tobacco and five of the ten brands of heated WP tobacco. The remaining five brands of heated WP tobacco contained benzene ranging from 73.6 ± 22.2 to 134.2 ± 19.6 ng/g. Although all ten brands of heated WP tobacco did not contain isoprene above the LOQ (8 ng/g), Brands T2 (blueberry and mint), T4 (passion fruit), and T9 (watermelon) of unheated WP tobacco contained isoprene at levels ranging from 8.3 ± 0.4 ng/g to 22.5 ± 0.5 ng/g. Acrolein was quantifiable in all heated WP tobacco products (43.0 ± 1.0 to 75.7 ± 9.3 ng/g) except for brands T9 (watermelon) and T10 (orange), while the levels were below the LOQ (40 ng/g) in all brands of unheated WP tobacco. FA levels in heated and unheated WP tobacco ranged from 2.6 ± 0.3 μg/g to 4.4 ± 0.7 μg/g and 0.2 ± 0.1 to 5.4 ± 0.2 μg/g, respectively.

The concentrations of B[a]P in unheated WP tobacco products ranged from below the LOQ (0.5 ng/g) to 1.9 ± 0.2 ng/g across the ten brands tested. B[a]P is at or below the LOQ for seven WP tobacco brands (T1 [chocolate and mint], T2 [blueberry and mint], T3 [orange and cherry], T6 [blueberry mint], T7 [apple], T9 [watermelon], and T10 [orange]). B[a]P quantities in heated WP products were similar to those found in unheated products and ranged from LOQ (0.5 ng/g) to 2.2 ± 0.2 ng/g. B[a]P was below the LOQ in 4 WP tobacco brands (T3 [orange and cherry], T7 [apple], T9 [watermelon], and T10 [orange]).

The quantities of NNN and NNK in unheated WP tobacco ranged from 2.7 ± 0.5 to 211.4 ± 4.1 ng/g and from 4.7 ± 0.5 to 298.3 ± 5.3 ng/g, respectively. Most unheated WP tobacco brands contained less than 50 ng/g for NNN and NNK except for the T9 (watermelon) brand. T9 brand is a nicotine gel that lacks tobacco leaves and necessary microbes to convert nicotine into NNN and NNK [56]. Therefore, no TSNA was detected above the LOQ of 5 ng/g in the T9 brand. The quantities of NNN and NNK in heated WP tobacco were comparable to those for unheated WP tobacco, which ranged from 17.7 ± 1.8 to 141.4 ± 30.2 ng/g for NNN and 6.5 ± 0.5 to 291.8 ± 35.5 ng/g for NNK. Neither NNN nor NNK was in the T9 brand, and NNN was not detected in six other WP tobacco brands (T1 [chocolate and mint], T2 [blueberry and mint], T4 [passion fruit], T5 [blueberry and mint], T6 [blueberry and mint], and T8 [peach]).

Catechol quantities in unheated and heated WP tobacco products ranged from 21.1 ± 1.1 to 50.5 ± 0.5 μg/g and from 40.9 ± 4.6 to 56.8 ± 5.5 μg/g, respectively, except for the T9 brand, where catechol was below the LOQ (0.7 μg/g) in both unheated and heated WP tobacco products. Furthermore, phenol quantities in unheated and heated WP tobacco products ranged from 0.6 ± 0.1 to 9.1 ± 0.1 μg/g and from 1.3 ± 0.1 to 15.9 ± 2.1 μg/g, respectively.

Supplementary Table S1 shows the recovered WP tobacco, and WP charcoal percentages ranged from 26.1% ± 4.3% to 63.5% ± 7.5% and from 58.2% ± 3.1% to 75.7% ± 1.0%, respectively. The T9 tobacco sample had the lowest recovery rate because it was a gel product; the sample was wet at the start of the experiment, but it dried out quickly afterward. It appeared that all the WP charcoal samples with a disc shape had a higher recovery rate than charcoal samples with a cube or finger shape. Unheated WP tobacco samples had an average pH of 4.76 ± 0.01, while the heated WP tobacco samples had an average pH of 5.33 ± 0.13 (Supplementary Methods). Nicotine was detected above or close to the LOQ (0.5 ng/g) in all WP tobacco brands except the T9 brand (Supplementary Methods and Table S2). Total nicotine quantities in unheated and heated WP tobacco ranged from 0.7 ± 0.0 to 3.1 ± 0.0 mg/g and from 0.3 ± 0.1 to1.0 ± 0.4 mg/g, respectively.

Trace metal As increased 33% - 127% in all heated compared to unheated WP tobacco products except for brands T4 (passion fruit) and T5 (blueberry mint), which were below the LOQ (0.1 μg/L). There were 78% - 198% and 14% - 203% increases, respectively, in Cd and Cr quantities observed in heated WP tobacco products compared to unheated products in all brands except T4 (passion fruit) and T9 (watermelon). Cd was not detected in the T9 (watermelon) brand, and the quantity of Cr was 23% lower in the heated product for the T4 (passion fruit) brand. Co was detected in all heated WP tobacco products, and a 91% - 168% increase of Co was measured in the heated products of T5 (blueberry and mint), T7 (apple), and T10 (orange) compared to the unheated products. Although Co was detected in all heated products except T9 (watermelon) brand, the quantities were below the LOQ (0.1 μg/L) in most unheated products, and therefore the % increase can only be determined for brands T5 (blueberry and mint, 112%), T7 (apple, 168%), and T10 (orange, 91%). Increases in Pb and Ni quantities of 59% - 518% and 47% - 117%, respectively, in heated WP tobacco products compared to unheated WP products were found in all WP tobacco brands except T9 (watermelon). Furthermore, a 37% - 93% increase in Se quantities in heated WP tobacco products compared to unheated WP products was only observed in T3 (orange and cherry) and T10 (orange) brands.

### 3.2. Humectants in WP Tobacco

Supplementary Table S3 shows the quantities of ethylene glycol, glycerol, and propylene glycerol in unheated WP tobacco ranged from below the LOQ (5 μg/g) to 1.5 ± 0.1 mg/g, from 211.1 ± 19.2 to 720.3 ± 5.7 mg/g, and from below the LOQ (4 μg/g) to 87.4 ± 2.9 mg/g, respectively. Ethylene glycol was below the LOQ for 4 WP tobacco brands (T1 [chocolate and mint], T8 [peach], T9 [watermelon], and T10 [orange]), and propylene glycol was below the LOQ for the T9 (watermelon) brand only (Supplementary Methods).

### 3.3. HPHCs in WPC Harcoal

Trace metals and B[a]P were investigated in the WP charcoal samples. B[a]P was found in all unburned WP charcoal products and ranged from 5.3 ± 0.6 to 95.0 ± 8.0 ng/g. B[a]P was not detected in any of the burned charcoal products. As quantities in unburned and burned charcoal products ranged from 91 to 3147 ng/g and from 129 to 7779 ng/g, respectively. A 42% - 147% increase in As quantities were found in all burned WP charcoal samples compared to the corresponding unburned WP charcoal samples except for brand C1 (beech wood, disc shape), which remained unchanged. Cd was only found in unburned and burned WP charcoal samples in brands C3 (instant and disc shape, 7% decrease) and C5 (natural and finger shape, 5% decrease). Increases in Cr (50% - 180%), Co (45% - 246%), Pb (20% - 197%), and Ni (68% - 202%) quantities of burned charcoal samples compared to the corresponding unburned samples were found in all WP charcoal brands. Increases in Se (58% - 105%) quantities were found in the burned samples for brands C4 (quicklight, disc shape) and C5 (natural and finger shape) compared to the unburned WP samples.

## 4. Discussion

This study investigated the impact and possible toxic risks to the environment of disposing of used WP tobacco and burned WP charcoal products by quantifying selected toxic chemical constituents in tobacco and charcoal before and after consumption. WP may be perceived as less harmful than cigarettes due to WP tobacco’s fruit flavors, sugary taste, and the belief that the smoke is filtered by the water. These factors may contribute to their appeal to young adults [2] [19]. Although the environmental impact of cigarette use is well documented [34] [57] [58], less is known about the environmental impact of WP use. This work does not focus on the HPHCs released in the smoke from WP tobacco products as this has been previously investigated [18] [39] [59].

In this work, we evaluated a series of WP tobacco products with different flavors (e.g., chocolate, mint, blueberry, orange, cherry, passion fruit, apple, peach, watermelon) as well as WP charcoal products with different materials (e.g., beech wood, coconut husks, instant, quicklight, natural) and shapes (e.g., disc, cube, finger), and quantified HPHCs present in these products pre- and post-consumption. For data analysis purposes, it would be useful to have a commercially available tobacco product that contains nicotine but does not have a traditional tobacco matrix for comparison because uptake of heavy metals by tobacco plant roots has been demonstrated [32]. To probe this tobacco matrix difference, we selected a nicotine gel for this investigation because it utilizes water-soluble polymeric materials for rapid nicotine release. We included a nicotine gel (Brand T9, watermelon flavor) as one of the ten tobacco products to evaluate and examine selected HPHCs and trace metals in the absence of tobacco leaves.

Table 1 and Table 2 show the yields of selected HPHCs in WP tobacco products under unheated (pre-consumption) and heated (post-consumption) conditions. Under ambient conditions, the quantification of acrolein, benzene, and isoprene in tobacco products has been a challenge due to the nature of their low boiling points (53°C, 81°C, and 34°C, respectively). This was particularly troublesome for tobacco products stored in non-sealed packaging containers, extraction efficiency with different organic solvents, and experiments conducted under a pressurized and ventilated environment.

The concentrations of acrolein and benzene ranged from 43.0 ± 1.0 ng/g to 75.7 ± 9.3 ng/g and 73.6 ± 30.1 to 134.2 ± 19.6 ng/g, respectively, in the heated tobacco samples. However, the concentrations of these analytes were found to be below LOQ in all the unheated tobacco samples. This suggests that both acrolein and benzene were primarily produced only during the consumption of tobacco samples, and a fraction of the overall quantities remained in the WP tobacco sample. Acrolein has been shown [60] to form from heating sugars; therefore, we expected to see acrolein in all WP tobacco samples after heating. However, acrolein quantities were below the LOQ in heated T9 (watermelon) and T10 (orange) tobacco samples; T9 was a gel, and the gel matrix may interfere with the heating process or contain less sugar content than other WP tobacco samples. Due to the relatively high volatility of carbonyl groups and hydrocarbons, it is reasonable to expect that acrolein and benzene may not remain in the heated WP tobacco. Interestingly, for unheated WP tobacco, the isoprene concentrations in seven of ten brands were below LOQ (8 ng/g), while three brands (T2 [blueberry and mint], T4 [passion fruit], and T9 [watermelon]) had levels ranging from 8.3 to 22.5 ng/g. However, for heated WP tobacco, the levels of isoprene in all ten brands were below LOQ. The observed low yields of isoprene may be due to its low boiling point (high volatility), and most of the isoprene produced during heating was transferred to the smoke phase.

We also observed low concentrations of catechol, phenol, FA, and B[a]P in the WP tobacco before and after heating, which may suggest that these HPHCs, which are typically produced during the consumption of WP tobacco, are readily transferred to the smoke phase, and their concentrations are minor in the consumed WP tobacco. Potential random errors may have contributed to uncertainties of measured results (e.g., FA), including heating temperature and heating behavior. Large uncertainties from repeated runsin waterpipe tobacco products have been observed by others [61]. Some of these HPHCs have been documented to have an adverse effect on aquatic biota and the environment [62] [63] [64].

The concentrations of TSNAs, such as NNN and NNK, remained consistent pre- and post-consumption. The potential environmental risks caused by TSNAs in the aquatic environment are still limited [65] [66]. We observed limited HPHCs in the consumed WP tobacco from brand T9 (*i.e*., isoprene, phenol, FA). It should be noted that brand T9 (watermelon) is a nicotine-containing gel and not a traditional WP tobacco product; therefore, no tobacco-derived HPHCs are expected.

We quantified humectants such as ethylene glycol, glycerol, and propylene glycerol in unheated WP tobacco products (Supplementary Table S3), and the results are comparable to what is reported in the literature [67] for other tobacco products except for glycerol (mean of ten brands, 371 ± 140 mg/g). However, higher levels of glycerol in WP tobacco are not unexpected as it makes it easier for manufacturers to minimize the harshness of the WP tobacco for use by the consumer. Furthermore, Schubert *et al*. demonstrated that an increase in the humectant levels in unburned tobacco lowers the temperature in the WP head during a smoking session, thereby decreasing carbonyl levels in the smoke produced [68].

Although brand T9 (watermelon) is advertised and labeled as a nicotine-containing gel, the nicotine content of the gel was evaluated for confirmation, and nicotine was not detected in either heated or unheated samples. It is possible that gel manufacturing precluded the presence of nicotine, or the nicotine degraded over time, given the nature of the hydrogel [69]. Using various extraction and analysis methods [70], including basic conditions (pH > 7) with 2N NaOH, resulted in detecting trace nicotine levels in the T9 (watermelon) brand of heated WP tobacco. However, these nicotine levels were an order of magnitude below the LOQ (5 ng/g) and thus could not be reliably quantified.

**Figure 2.**
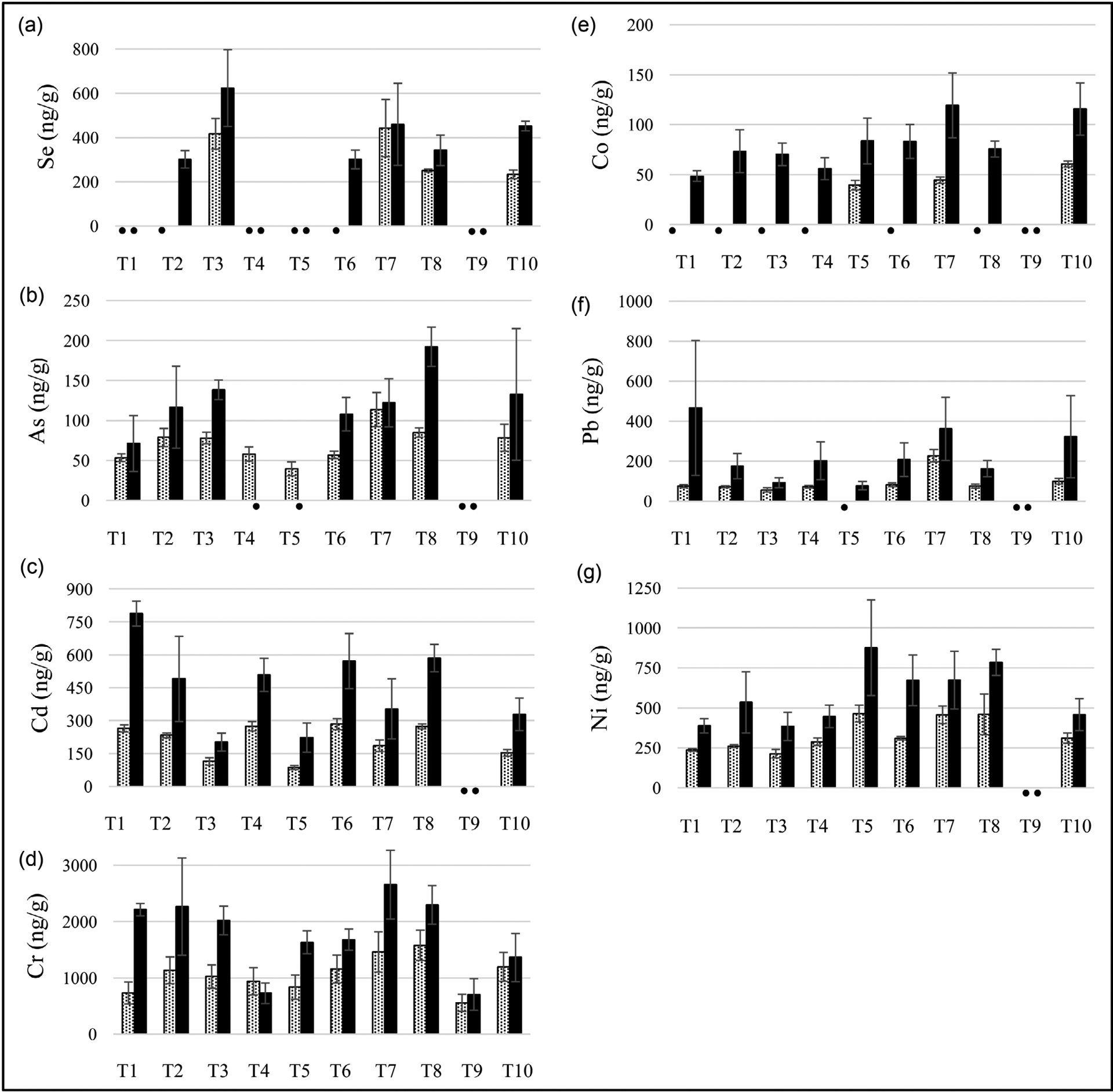
Comparison of trace metals in unheated (gray bars) and heated (black bars) WP tobacco solid waste. (•) meansbelow the limit of quantification. LOQ for Se (0.5 μg/L); As (0.1 μg/L), Cd (0.1 μg/L), Cr (0.5 μg/L), Co (0.1 μg/L), Pb (0.1 μg/L), Ni (0.1 μg/L). Error bars indicate standard deviations.

The pH of a tobacco product is known to affect the amount of unprotonated nicotine (the more readily absorbed form) [71] and influence TSNA formation [72]. An increase in pH in the WP tobacco product after heating was observed in all brands (Supplementary Table S2). This increase in alkalinity could lower metal ion solubility [73] [74] and cause metal ions to precipitate, accumulate, and remain in the tobacco products over time. The concentration of trace metals as a function of WP tobacco brands before and after heating is shown in Figure 2. Heavy metals are known to impact environmental health negatively [75]. All metals except for Cr were shown to increase in concentration in the remaining tobacco after heating. Increases in metal concentration after product heating may be a result of lower total tobacco mass. Interestingly, Cr is the only trace metal found above the LOQ for both unheated and heated conditions for brand T9; this is likely due to the absence of tobacco leaves that often carry heavy metal [24]. The T8 brand contained the most As, resulting in a 127% increase from 85 ± 6 ng/g before heating to 192 ± 25 ng/g after heating.

**Figure 3.**
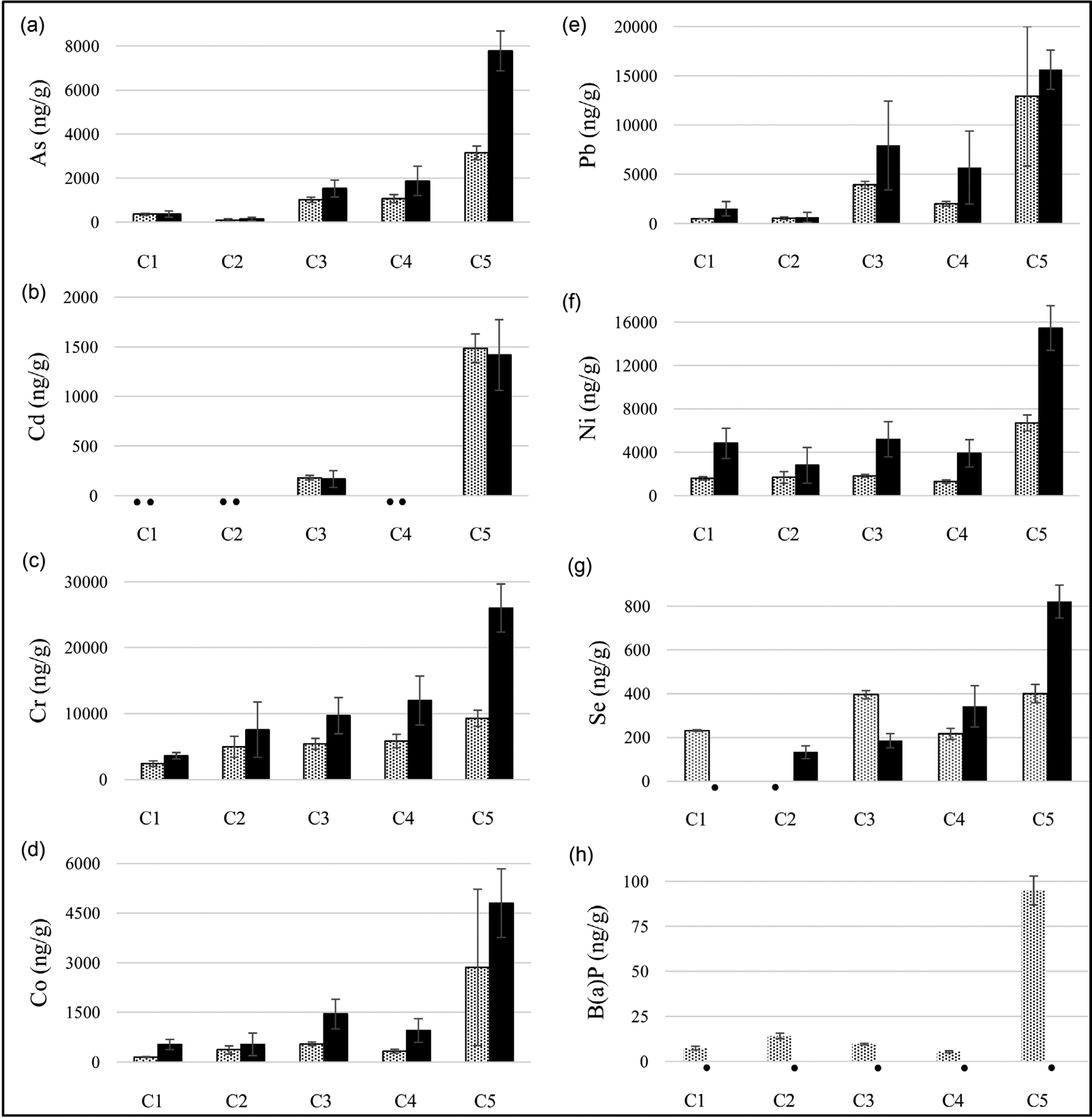
Comparison of trace metals and B[a]P in unburned (gray bars) and burned (black bars) WP charcoal solid waste. (•) means below the limit of quantification. LOQ for As (0.1 μg/L), Cd (0.1 μg/L), Cr (1 μg/L), Co (0.1 μg/L), Pb (0.1 μg/L), Ni (0.1 μg/L), Se (0.5 μg/L), B[a]P (0.5 ng/g).

It was difficult to accurately extract volatile HPHCs from WP charcoal samples because activated charcoal acts like a sponge and can readily soak up organic compounds. Therefore, only inorganic trace metals and B[a]P are reported in this study. Figure 3 shows the overall trace metals and B[a]P levels in all WP charcoal brands under unburned and burned conditions. Interestingly, the charcoal sample profile follows the same trend as WP tobacco samples in Figure 2 (increasing metal concentration upon heating). For brands C3 (instant, disc shape), C4 (quicklight, disc shape), and C5 (natural, disc shape), the concentrations of trace metals increased by 52% - 147% (As), 79% - 180% (Cr), 130% - 201% (Ni), and 58% - 105% (Se) in the burned sample, but the concentrations remained the same in brands C1 (beech wood, disc shape) and C2 (coconut husks, cube shape). This finding is interesting because consumers consider that WP charcoal made from coconut and natural woods to be less harmful than synthetic WP charcoals due to limited additives being added to the product [76]. This result may have occurred because the metal concentrations were relatively low at or below the LOQ. Since it can be assumed that the moisture content of WP charcoal is relatively low, humectants are not typically added to WP charcoal products. Therefore, the increase of trace metal quantities is likely due to something other than pH changes.

A WP is often coated with a layer of metallic paint for aesthetic purposes, so coating substances may be depleted and introduced into the charcoal sample during the burning process. However, a more plausible explanation is that all tobacco and charcoal samples experience a mass loss due to heating (volatilization or combustion). Under these temperature conditions, it can be assumed that trace metals are nonvolatile; they most likely remain in the heated products. The decrease in total product mass yielded an apparent increase in metal concentrations (except As in brands T4 [passion fruit] and T5 [blueberry and mint]).

Table 3 shows the comparison between trace metal yield range results of different tobacco product solid waste (*i.e*., WP tobacco, little cigar, cigarette, smokeless, WP charcoal). The yield results from the WP tobacco brand T9 (nicotine gel) were excluded due to the absence of tobacco leaves. Interestingly, all our metals except for Ni in WP tobacco were shown to have higher concentration ranges (before heating) than observed by Saadawi *et al*. [77]. This difference may be due to the variability in the non-tobacco ingredients in the WP tobacco mixture. Tobacco molasses is a common non-tobacco ingredient used to manufacture WP tobacco mixtures and has been shown to contain high concentrations of trace metals [78] [79] [80]. Generally, metal concentration ranges in WP tobacco before heating appears lower than those reported for the little cigar, cigarette, and smokeless tobacco product solid waste [77] [81] [82] [83] [84] [85], which are not unexpected as flavored WP tobacco mixture is composed of approx. 30% tobacco and 70% non-tobacco ingredients [19].

Currently, the trace metal yield results generated under heating conditions in other tobacco product solid waste (tobacco-derived) are limited in the literature, making the comparison of metal concentration ranges relatively challenging. This may be worth exploring in the future, particularly with solid waste from heated tobacco products (HTPs). Our metal concentration ranges in WP charcoal before burning were in the same order of magnitude as those reported by others [76] [31]. However, it appeared that a greater enhancement of metal concentrations after burning in WP charcoal solid waste was observed by Saadawi *et al*. [31], which suggests that there may be other factors contributing to the metal yields during the combustion process; these other factors may include the WP apparatus materials (e.g., WP head, metallic coating, aluminum foil), as they all had direct contact with the WP charcoal solid waste during the burning process.

Figure 3(h) shows that B[a]P was not detected in all WP charcoal samples after heating but was detected before heating (7.3, 14.1, 9.5, 5.4, and 95 ng/g for C1, C2, C3, C4, C5, respectively). The results of B[a]P concentrations (before heating) are in good agreement with B[a]P concentrations found in raw WP charcoal by others [48] [86], which suggests that B[a]P is likely transferred to the tobacco smoke during the WP smoking session. WP charcoal brand C5 (natural and finger shape) contains substantially higher quantities of heavy metals and B[a]P compared to other brands investigated, and this result shows that the labeling of WP charcoal could be misleading (ie, natural) and potentially give consumers a false sense of security when using WP tobacco products.

## 5. Conclusion

In this study, the concentrations of selected HPHCs (acrolein, benzene, isoprene, catechol, phenol, FA, B[a]P, NNN, and NNK) and selected trace elements (As, Cd, Cr, Pb, Se, Co, Ni) were investigated in ten WP tobacco products with flavors and five WP charcoal products with different materials and shapes. No noticeable trend is observed among the WP tobacco flavors. However, WP charcoal brand C5 (natural and finger shape) contained substantially higher quantities of heavy metals and B[a]P than other brands investigated. This study demonstrates that trace metal residues are present in unheated and unburned WP tobacco and charcoal and are present at higher concentrations after consumer use and thus could have an environmental impact. Results indicate that Cr, Pb, and Ni are the most abundant metals in most WP charcoal brands we investigated. We also demonstrated that WP tobacco and WP charcoal products contained substantial quantities of B[a]P before heating or burning, but B[a]P was not detected in the spent charcoal after consumption. This study characterizes the environmental harms of burned WP tobacco and charcoal products and their potential impact on environmental health. Based on the current data and use patterns, the environmental impact for WP may seem small. However, more studies are needed to estimate the magnitude of the environmental impact of WP tobacco use.

## Supplementary Material

1

## Figures and Tables

**Table 1.** Quantification of HPHCs in unheated WP tobacco solid waste.

WP Tobacco Brands	^a^Acrolein	^a^Benzene	^a^Isoprene	^b^Catechol	^b^Phenol	^b^FA	^c^B[a]P	^c^NNK	^c^NNN
T1 Chocolate/Mint	BLOQ	BLOQ	BLOQ	31.4 ± 0.8	1.9 ± 0.1	0.2 ± 0.1	BLOQ	4.7 ± 0.5	BLOQ
T2 Blueberry/Mint	BLOQ	BLOQ	8.3 ± 0.4	36.1 ± 0.6	1.2 ± 0.2	1.2 ± 0.2	BLOQ	9.0 ± 1.8	5.0 ± 0.9
T3 Orange/Cherry	BLOQ	BLOQ	BLOQ	50.5 ± 0.5	1.6 ± 0.1	2.0 ± 0.9	BLOQ	37.0 ± 2.0	26.6 ± 1.9
T4 Passion Fruit	BLOQ	BLOQ	22.5 ± 0.5	40.6 ± 1.5	5.6 ± 0.4	1.0 ± 0.1	1.5 ± 0.3	6.1 ± 0.3	BLOQ
T5 Blueberry/Mint	BLOQ	BLOQ	BLOQ	49.8 ± 1.9	3.4 ± 0.2	5.4 ± 0.2	1.9 ± 0.2	5.3 ± 0.1	BLOQ
T6 Blueberry/Mint	BLOQ	BLOQ	BLOQ	25.9 ± 3.6	1.7 ± 0.7	2.8 ± 0.1	BLOQ	5.9 ± 0.5	3.8 ± 0.7
T7 Apple	BLOQ	BLOQ	BLOQ	45.2 ± 0.9	9.1 ± 0.1	0.3 ± 0.1	BLOQ	38.3 ± 1.5	22.3 ± 1.2
T8 Peach	BLOQ	BLOQ	BLOQ	36.6 ± 0.8	5.5 ± 0.2	1.8 ± 0.2	1.1 ± 0.3	7.4 ± 0.6	2.7 ± 0.5
T9 Watermelon	BLOQ	BLOQ	10.5 ± 0.5	BLOQ	0.6 ± 0.1	2.8 ± 0.2	BLOQ	BLOQ	BLOQ
T10 Orange	BLOQ	BLOQ	BLOQ	21.1 ± 1.1	2.3 ± 0.2	1.6 ± 0.6	BLOQ	298.3 ± 5.3	211.4 ± 4.1

Note.

a Acrolein, Benzene, Isoprene values are presented in ng/g (mean ± s.d., N = 7 per brand). BLOQ means below the limit of quantification, LOQ for acrolein, benzene, and isopresent are 40 ng/g, 56 ng/g, and 8 ng/g, respectively.

b Catechol, Phenol, FA values are presented in μg/g (mean ± s.d., N = 7 per brand). BLOQ means below the limit of quantification, LOQ for catechol, phenol, and FA are 0.7 μg/g, 0.1 μg/g, and 0.03 μg/g, respectively.

c B[a]P, NNK, NNN values are presented in ng/g (mean ± s.d., N = 7 per brand). BLOQ means below the limit of quantification, LOQ for B[a]P, NNK, and NNN are 0.5 ng/g, 5 ng/g, and 5 ng/g, respectively.

**Table 2.** Quantification of HPHCs in heated WP tobacco solid waste.

WP Tobacco Brands	^a^Acrolein	^a^Benzene	^a^Isoprene	^b^Catechol	^b^Phenol	^b^FA	^c^B[a]P	^c^NNK	^c^NNN
T1 Chocolate/Mint	75.7 ± 9.3	BLOQ	BLOQ	40.9 ± 4.6	11.5 ± 1.4	4.4 ± 0.7	1.2 ± 0.1	6.5 ± 0.5	BLOQ
T2 Blueberry/Mint	53.7 ± 7.9	BLOQ	BLOQ	52.8 ± 12.2	8.1 ± 1.1	4.1 ± 1.0	1.3 ± 0.2	10.4 ± 0.9	BLOQ
T3 Orange/Cherry	53.9 ± 16.2	BLOQ	BLOQ	43.2 ± 3.0	7.5 ± 0.7	2.6 ± 0.3	BLOQ	45.7 ± 6.7	17.7 ± 1.8
T4 Passion Fruit	65.6 ± 14.4	73.6 ± 30.1	BLOQ	44.8 ± 3.6	6.9 ± 0.3	3.5 ± 0.5	2.2 ± 0.2	7.7 ± 0.7	BLOQ
T5 Blueberry/Mint	65.6 ± 10.6	95.9 ± 22.4	BLOQ	45.7 ± 8.8	13.0 ± 1.3	3.4 ± 0.3	1.5 ± 0.1	7.2 ± 1.3	BLOQ
T6 Blueberry/Mint	43.0 ± 1.0	96.6 ± 30.6	BLOQ	56.8 ± 5.5	7.5 ± 1.3	2.9 ± 0.2	1.3 ± 0.2	12.9 ± 1.9	BLOQ
T7 Apple	51.5 ± 2.0	73.6 ± 22.2	BLOQ	43.7 ± 6.5	15.9 ± 2.1	2.9 ± 0.5	BLOQ	44.4 ± 4.1	26.6 ± 3.8
T8 Peach	43.8 ± 3.2	BLOQ	BLOQ	49.8 ± 9.8	8.2 ± 0.5	4.0 ± 0.6	1.6 ± 0.1	15.0 ± 2.6	BLOQ
T9 Watermelon	BLOQ	BLOQ	BLOQ	BLOQ	1.3 ± 0.1	2.9 ± 1.4	BLOQ	BLOQ	BLOQ
T10 Orange	BLOQ	134.2 ± 19.6	BLOQ	48.3 ± 7.9	6.0 ± 0.6	3.0 ± 0.3	BLOQ	291.8 ± 35.5	141.4 ± 30.2

Note.

a Acrolein, Benzene, Isoprene values are presented in ng/g (mean ± s.d., N = 7 per brand). BLOQ means below the limit of quantification, LOQ for acrolein, benzene, and isopresent are 40 ng/g, 56 ng/g, and 8 ng/g, respectively.

b Catechol, Phenol, FA values are presented in μg/g (mean ± s.d., N = 7 per brand). BLOQ means below the limit of quantification, LOQ for catechol, phenol, and FA are 0.7 μg/g, 0.1 μg/g, and 0.03 μg/g, respectively.

c B[a]P, NNK, NNN values are presented in ng/g (mean ± s.d., N = 7 per brand). BLOQ means below the limit of quantification, LOQ for B[a]P, NNK, and NNN are 0.5 ng/g, 5 ng/g, and 5 ng/g, respectively.

**Table 3.** Comparison of metal concentration ranges in various tobacco product solid waste.

Product Type		^a^As	^a^Cd	^a^Cr	^a^Co	^a^Pb	^a^Ni	^a^Se
*Tobacco*								
WP Tobacco (our results)	BH	40 – 114	87 – 285	732 – 1581	<0.1 – 61	<0.1 – 227	213 – 464	<0.5 – 443
	AH	<0.1 – 192	203 – 788	728 – 2657	48 – 119	77 – 466	384 – 875	<0.5 – 624
WP Tobacco [77]	BH	10 – 40	100 – 270	150 – 370		<1 – 70	140 – 640	2 – 14
	AH							
Little Cigar [81]	BH	120 – 660	752 – 1740	880 – 6460	650 – 1000	460 – 1230	1500 – 4370	90 – 369
	AH							
Cigarette [82]	BH	250 – 1070	20 – 50	30 – 1580		600 – 7930	370 – 1180	180 – 340
	AH							
Cigarette [83]	BH		243 – 795		1233 – 4066	2500 – 27,330		
	AH							
Smokeless [84]	BH		10 – 170	2770 – 11,400	10 – 30	0 – 2480	20 – 70	
	AH							
Smokeless [85]	BH		1750 – 1950	15020 – 16,380		1560 – 1700	1380 – 1770	
	AH							
*Charcoal*								
WP Charcoal (our results)	BB	91 – 3147	<0.1 – 1485	2399 – 9287	153 – 2857	498 – 12,922	1298 – 6699	<0.5 – 400
	AB	356 – 7779	<0.1 – 1417	3609 – 26,029	530 – 4799	638 – 15,621	2800 – 15,440	<0.5 – 821
WP Charcoal [76]	BB		110 – 1530	2350 – 2520		2820 – 9090		
	AB							
wp Charcoal [31]	BB	15 – 10,300	3 – 2100	161 – 8320	124 – 10,000	95 – 55,600	274 – 14,000	
	AB	377 – 57,200	2 – 3360	7380 – 142,000	1180 – 24,600	2440 – 81,200	6070 – 204,000	366 – 3330

Note.

a As, Cd, Cr, Co, Pb, Ni, Se all values are presented in ng/g;

BH (before heating); AH (after heating); BB (before burning); AB (after burning).
