# Supplementary material for: Levels of Chemical Toxicants in Waterpipe Tobacco and Waterpipe Charcoal Solid Waste
Source: J Environ Prot (Irvine, Calif). Author manuscript; Available in PMC 2021 Dec 7. (PMC8649808; doi:10.4236/jep.2021.1211054)
Supplement: 1 [file NIHMS1760365-supplement-1.pdf]

Supplementary

Table S1. Comparison of recovered WP tobacco and WP charcoal solid waste.

WP Tobacco Brands	[a]Recovery (%)	WP Charcoal Brands	[a]Recovery (%)
T1 Chocolate/Mint	55.2 ± 6.1	C1 Beech Wood/Disc Shape	72.5 ± 0.8
T2 Blueberry/Mint	54.4 ± 5.0	C2 Coconut Husks/Cube Shape	69.6 ± 3.2
T3 Orange/Cherry	47.2 ± 7.2	C3 Instant/Disc Shape	72.4 ± 1.6
T4 Passion Fruit	50.9 ± 5.3	C4 Quicklight/Disc Shape	75.7 ± 1.0
T5 Blueberry/Mint	63.5 ± 7.5	C5 Natural/Finger Shape	58.2 ± 3.1
T6 Blueberry/Mint	53.7 ± 4.7		
T7 Apple	44.8 ± 4.1		
T8 Peach	51.6 ± 3.8		
T9 Watermelon	26.1 ± 4.3		
T10 Orange	53.1 ± 8.3		

Note. [a]Recovery (%) values are presented in %, mean ± s.d., N = 7 per brand.

Table S2. Comparison of pH and nicotine changes in WP tobacco solid waste.

WP Tobacco Brands	[a]Unheated pH	[a]Heated pH	[b]Unheated Total Nicotine	[b]Heated Total Nicotine	[b]Unheated Free Nicotine	[b]Heated Free Nicotine
T1 Chocolate/Mint	4.5 ± 0.0	5.2 ± 0.1	0.9 ± 0.0	BLOQ	BLOQ	BLOQ
T2 Blueberry/Mint	4.7 ± 0.0	5.2 ± 0.0	0.7 ± 0.0	BLOQ	BLOQ	BLOQ
T3 Orange/Cherry	4.4 ± 0.0	5.0 ± 0.1	1.5 ± 0.0	0.8 ± 0.2	BLOQ	BLOQ
T4 Passion Fruit	4.8 ± 0.0	5.3 ± 0.1	0.8 ± 0.2	BLOQ	BLOQ	BLOQ
T5 Blueberry/Mint	4.3 ± 0.0	5.1 ± 0.2	0.7 ± 0.0	BLOQ	BLOQ	BLOQ
T6 Blueberry/Mint	4.9 ± 0.0	5.2 ± 0.1	1.0 ± 0.0	BLOQ	BLOQ	BLOQ
T7 Apple	4.9 ± 0.0	5.2 ± 0.1	1.8 ± 0.0	0.8 ± 0.2	BLOQ	BLOQ
T8 Peach	4.7 ± 0.0	5.2 ± 0.1	0.7 ± 0.0	BLOQ	BLOQ	BLOQ
T9 Watermelon	5.2 ± 0.0	5.6 ± 0.2	BLOQ	BLOQ	BLOQ	BLOQ
T10 Orange	5.3 ± 0.0	6.2 ± 0.2	3.1 ± 0.0	1.0 ± 0.4	BLOQ	BLOQ

Note. [a]Unheated and Heated pH values are presented in mean ± s.d., N = 7 per brand. [b]Unheated, Heated, Total or Free Nicotine values are presented in mg/g (mean ± s.d., N = 7 per brand). BLOQ means below the limit of quantification and the LOQ for nicotine is 0.5 ng/g.

Table S3. Comparison of humectant in unheated WP tobacco solid waste.

WP Tobacco Brands	[a]Ethylene Glycol	[b]Propylene Glycol	[c]Glycerol
T1 Chocolate/Mint	BLOQ	33.3 ± 0.1	377.2 ± 3.7
T2 Blueberry/Mint	0.5 ± 0.0	6.4 ± 0.2	386.7 ± 6.5
T3 Orange/Cherry	0.4 ± 0.0	7.1 ± 0.2	279.3 ± 8.1
T4 Passion Fruit	0.5 ± 0.0	16.8 ± 0.4	381.5 ± 4.5
T5 Blueberry/Mint	1.5 ± 0.1	87.4 ± 2.9	384.2 ± 5.3
T6 Blueberry/Mint	1.0 ± 0.1	37.0 ± 0.4	378.2 ± 3.6
T7 Apple	0.4 ± 0.0	13.1 ± 0.7	226.1 ± 5.6
T8 Peach	BLOQ	21.4 ± 0.4	370.1 ± 6.2
T9 Watermelon	BLOQ	1.1 ± 0.0	720.3 ± 5.7
T10 Orange	BLOQ	BLOQ	211.1 ± 19.2

Note. [a]Ethylene Glycol values are presented in mg/g (mean ± s.d., N = 7 per brand). BLOQ means below the limit of quantification, LOQ for ethylene glycol (4 μg/g). [b]Propylene Glycol values are presented in mg/g (mean ± s.d., N = 7 per brand). BLOQ means below the limit of quantification, LOQ for propylene glycol (5 μg/g). [c]Glycerol values are presented in mg/g (mean ± s.d., N = 7 per brand). LOQ for glycerol (0.76 mg/g).

Supplementary Methods

GC-FID Analysis of Nicotine

Nicotine in WP tobacco samples (1 g each) was extracted using methyl-tert-butyl ether (MTBE) and isopropanol (IPA) and analyzed using GC (Agilent 6890) with a flame ionization detector (GC-FID) [70] and a fused silica capillary column (DB-WAX 15-m x 0.53-mm ID, 1.0 µm film thickness, Agilent 125-7012). Quinoline (CAS 91-22-5, 10 mg/mL) and nicotine (CAS 54-11-5, 50 mg/mL) stock solutions were diluted and used as calibration standards. The GC parameters were set as follows: column oven temperature of 65˚C was held for 2 min, and then ramped to 85˚C at a rate of 4˚C/min and held constant for 2 min. The oven temperature was ramped up again to 230˚C at a rate of 20˚C/min and held constant for another 2 min. The inlet and detector temperatures were set at 230˚C and 275˚C, respectively. Helium was used as the carrier gas at a column flow rate of 3 mL/min.

GC-FID Analysis of Humectants

Glycerol, propylene glycol, and ethylene glycol in unheated and heated WP tobacco samples (4 g of sample each) were extracted into 50 mL of methanol. 200 µL of 1,4-butanediol (500 mg/mL) was added and used as the internal standard. The extraction solution was shaken for 30 min on a shaker, and the liquid was decanted into a 50 mL polypropylene centrifuge tube and centrifuged for 2 min at 2000 rpm. After centrifugation, 1 mL of extraction solution is filtered through a 0.45 µm polytetrafluoroethylene (PTFE) syringe filter into a vial for analysis using GC-FID (Agilent 6890) [68]. The GC parameters were set as follows: column oven temperature of 50˚C was held for 2 min, and then ramped to 220˚C at a rate of 30˚C/min and held constant for 6 min. The split inlet and detector temperatures were set at 220˚C and 260˚C, respectively. The split ratio was 20:1, and helium was used as the carrier gas at a column flow rate of 1 mL/min. The column used was SOLGEL-WAX 30 m × 0.25 mm × 0.25 µm.

Determination of pH in heated and unheated tobacco

The pH of unheated and heated WP tobacco samples was determined using a glass combination electrode and conditions as previously described [87]. Unheated or heated tobacco samples (1 g of solid sample) were added into a 50 mL beaker followed by the addition of 10 mL of distilled water. pH measurements were acquired at 5, 15, and 30 mins after adding the water. The rearranged Henderson-Hasselbalch equation (Equation (1)) was used to calculate the free-base nicotine quantities:

$$\text{Nicotine}_{\text{free}} = \frac{\left[10^{\text{pH}-8.02}\right]\text{Nicotine}_{\text{total}}}{1+\left[10^{\text{pH}-8.02}\right]} \quad (1)$$
